# Sildenafil Reduces Expression and Release of IL-6 and IL-8 Induced by Reactive Oxygen Species in Systemic Sclerosis Fibroblasts

**DOI:** 10.3390/ijms21093161

**Published:** 2020-04-30

**Authors:** Luigi Di Luigi, Paolo Sgrò, Guglielmo Duranti, Stefania Sabatini, Daniela Caporossi, Francesco Del Galdo, Ivan Dimauro, Cristina Antinozzi

**Affiliations:** 1 Unit of Endocrinology, Department of Movement, Human and Health Sciences, University of Rome “Foro Italico”, 00135 Rome, Italy; luigi.diluigi@uniroma4.it (L.D.L.); paolo.sgro@uniroma4.it (P.S.); 2 Unit of Biochemistry and Molecular Biology, Department of Movement, Human and Health Sciences, University of Rome “Foro Italico”, 00135 Rome, Italy; guglielmo.duranti@uniroma4.it (G.D.); stefania.sabatini@uniroma4.it (S.S.); 3 Unit of Biology and Genetic, Department of Movement, Human and Health Sciences, University of Rome “Foro Italico”, 00135 Rome, Italy; daniela.caporossi@uniroma4.it (D.C.); ivan.dimauro@uniroma4.it (I.D.); 4 Division of Rheumatic and Musculoskeletal Diseases, Leeds Institute of Molecular Medicine, University of Leeds, Leeds LS2 9JT, UK

**Keywords:** systemic sclerosis, oxidative stress, inflammation, PDE5 inhibitors

## Abstract

Oxidative stress linked to vascular damage plays an important role in the pathogenesis of systemic sclerosis (SSc). Indeed, vascular damage at nailfold capillaroscopy in patients with Raynaud’s Phenomenon (RP) is a major risk factor for the development of SSc together with the presence of specific autoantiobodies. Here, we investigated the effects of the phosphodiesterase type 5 inhibitor (PDE5i) sildenafil, currently used in the management of RP, in modulating the proinflammatory response of dermal fibroblasts to oxidative stress in vitro. Human fibroblasts isolated from SSc patients and healthy controls were exposed to exogenous reactive oxygen species (ROS) (100 µM H_2_O_2_), in the presence or absence of sildenafil (1 µM). Treatment with sildenafil significantly reduced dermal fibroblast gene expression and cellular release of IL-6, known to play a central role in the pathogenesis of tissue damage in SSc and IL-8, directly induced by ROS. This reduction was associated with suppression of STAT3-, ERK-, NF-κB-, and PKB/AKT-dependent pathways. Our findings support the notion that the employment of PDE5i in the management of RP may be explored for its efficacy in modulating the oxidative stress-induced proinflammatory activation of dermal fibroblasts in vivo and may ultimately aid in the prevention of tissue damage caused by SSc.

## 1. Introduction

Systemic sclerosis (SSc) is a rare systemic disease characterized by autoimmune-driven inflammation, vasculopathy, and tissue fibrosis affecting the skin and internal organs. Fibrosis is the most prominent clinical feature of SSc and the major cause of mortality [1]. This process is led by excessive fibroblast activation and extra-cellular matrix deposition, which in turn causes multi-organ dysfunction [2]. Data from several groups demonstrated the pivotal role of oxidative stress in SSc pathogenesis and internal organ fibrogenesis, highlighting the interplay of an altered redox-state and abnormal activation of pro-inflammatory pathways [3,4]. Indeed, lymphocytes, T helper cells (i.e., 1 and 17), B-cell activation, and proinflammatory cytokines participate in the tissue damage caused by SSc through the overproduction of reactive oxygen species (ROS) [5]. In this regard, multiple lines of evidence indicate that interleukin (IL)-8 and IL-6 are critical in SSc pathogenesis [5,6,7]. Both interleukins are normally elevated in the blood of SSc patients, and their production/secretion is strongly sustained by immune cells and fibroblasts [5]. Importantly, monoclonal antibody-mediated blocking of the IL-6 pathway has been explored and has shown some benefit in preventing progression of lung fibrosis in SSc [8,9,10,11].

Two independent studies have shown that the phosphodiesterase inhibitors (PDEi) inhibit production and release of IL-6 and IL-8 in human peripheral blood leukocytes and cardiomyocytes subjected to inflammatory stimuli [12,13].

Sildenafil is a PDE5i routinely used for the management of Raynaud’s phenomenon, which is in turn the main clinical risk factor for developing SSc in people with specific Antinuclear Antibodies (ANA). Here, we set out to determine the effects of sildenafil on gene expression and release of IL-6 and IL-8 in cell cultures of human dermal fibroblasts (Hfb), which are thought to be among the key cellular elements in the pathogenesis of SSc.

## 2. Results

### 2.1. Sildenafil Inhibited Secretion and Gene Transcription of IL-6 and IL-8 Induced by Hydrogen Peroxide

**Figure 1:**
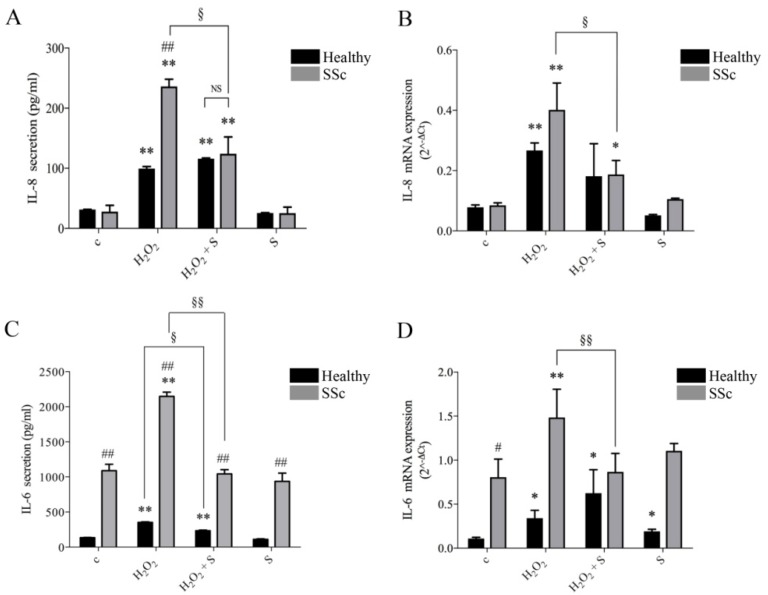
Supernatants (**A**,**C**) and RNAs extracted (**B**,**D**) from human healthy (black columns) and SSc (grey columns) fibroblast cultures exposed to H_2_O_2_ (100 µM, 24 h) with or without a pre-treatment with sildenafil (1 µM) were analyzed for IL-6 and IL-8 contents. Data are presented as the means ± SEM (*n* = 3). Statistical significance was determined using ANOVA with Bonferroni’s post-hoc test. * *p* < 0.05 and ** *p* < 0.01 vs. relative control within group; ^#^ *p* < 0.05 and ^##^ *p* < 0.01 vs. corresponding treatment between groups; ^§^ *p* < 0.05; ^§§^ *p* < 0.01. c, control group; S, sildenafil; NS, not significant.

H_2_O_2_ treatment induced strong release of IL-8 in dermal fibroblasts as expected (99.3 ± 3.5 vs. 31.4 ± 0.3 pg/mL basal levels) (Figure 1A). Dermal fibroblasts from SSc had a similar basal level but showed more than two-fold higher induction following H_2_O_2_ treatment (236.1 ± 12.0 pg/mL, *p* < 0.01). Pre-treatment with sildenafil suppressed the aberrant response of SSc cells, resulting in levels similar to healthy control fibroblasts (124.1 ± 28.0 pg/mL). Interestingly, sildenafil did not affect basal levels or the response of healthy control fibroblasts to H_2_O_2_ (Figure 1A). These results were paralleled at the gene transcription level. In particular, the pro-oxidant environment induced significant increases in IL-8 gene expression in healthy (control (c) vs. H_2_O_2_: IL-8, 0.1 ± 0.0 vs. 0.3 ± 0.0, *p* < 0.01) or in SSc fibroblasts (c vs. H_2_O_2_: IL-8, 0.1 ± 0.0 vs. 0.4 ± 0.2, *p* < 0.01). The concomitant presence of sildenafil in the culture medium reduced the effects of H_2_O_2_ on the gene expression of IL-8 in both experimental groups, healthy (H_2_O_2_ + S: 0.2 ± 0.2, *p* > 0.05) and SSc fibroblasts (H_2_O_2_ + S: 0.2 ± 0.1, *p* < 0.05). The presence of sildenafil did not produce any significant change in IL-8 expression in both cell lines (Figure 1B).

Supernatants from SSc fibroblasts secreted almost 10 times more IL-6 than healthy controls under basal conditions (1089.0 ± 91.0 pg/mL vs. 134.7 ± 3.7 pg/mL in healthy control fibroblasts, *p* < 0.01). The difference remained significant following exposure to H_2_O_2_, with the concentration rising to 351.8 ± 8.6 pg/mL for healthy control cells and 2148.0 ± 59.3 pg/mL for SSc cells (*p* < 0.01, Figure 1C). The pre-treatment with sildenafil reduced IL-6 secretion in both experimental groups (healthy: 233.0 ± 10.2 pg/mL; SSc: 1043.0 ± 60.0 pg/mL). Interestingly, sildenafil did not affect the basal levels of IL-6 secretion in either experimental group (Figure 1A,B). At the gene expression level, IL-6 showed increased basal levels in SSc fibroblasts, consistent with previous reports [14] (healthy vs. SSc: 0.1 ± 0.0 vs. 0.8 ± 0.4, *p* < 0.05) (Figure 1D).

The exposure to H_2_O_2_ induced a significant increase in IL-6 transcripts in both healthy (c vs. H_2_O_2_: 0.11 ± 0.02 vs. 0.3 ± 0.1, *p* < 0.05) and SSc fibroblasts (c vs. H_2_O_2_: 0.8 ± 0.4 vs. 1.5 ± 0.6, *p* < 0.01). Similar to IL-8, the presence of sildenafil in the culture medium completely inhibited the effect of ROS on IL-6 gene expression in SSc fibroblasts (*p* > 0.05). Interestingly sildenafil induced a slight but statistically significant increase in IL-6 expression in fibroblasts from healthy subjects (c vs. S: 0.11 ± 0.02 vs. 0.19 ± 0.03, *p* < 0.05) (Figure 1D).

### 2.2. Sildenafil Suppressed the Activation of Intracellular Pathways Induced by the Pro-Oxidant Condition in Healthy and SSc Fibroblasts

**Figure 2:**
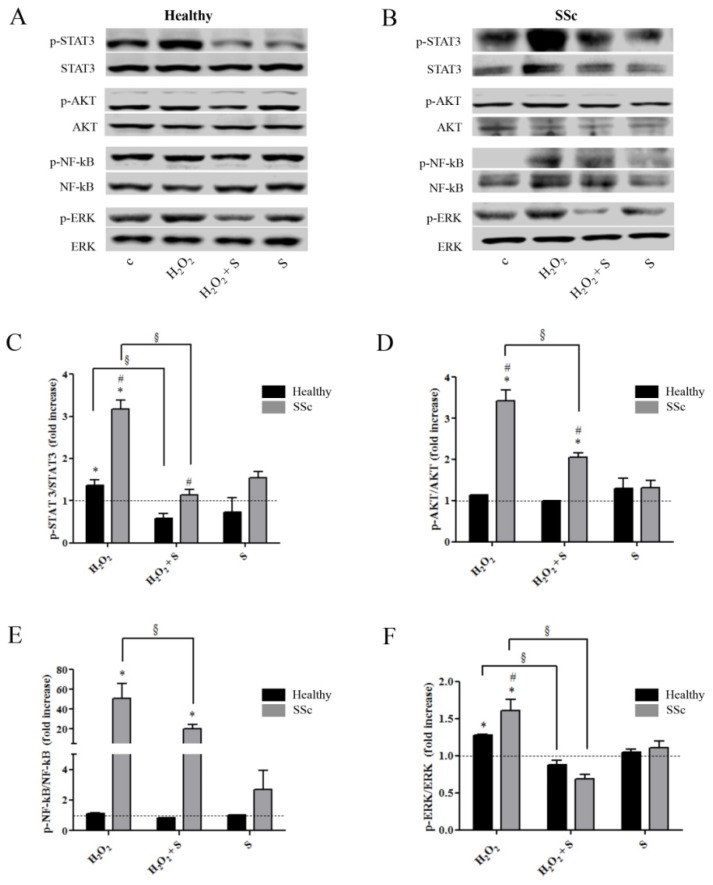
Representative western blot images of proteins analyzed in (**A**) healthy and (**B**) SSc fibroblasts. Proteins extracted from human healthy (black columns) and SSc (grey columns) fibroblasts exposed to H_2_O_2_ (100 µM, 24 h) with or without a pre-treatment with S (1 µM) were immunoblotted with antibodies against the total and phosphorylated form of (**C**) STAT3, (**D**) AKT, (**E**) NF-κB, and (**F**) ERK. Bars of the histogram show the ratio between the total and phosphorylated form of protein targets. Statistical significance was determined using ANOVA with Bonferroni’s post-hoc test. The dotted line indicates control levels. * *p* < 0.05 vs. relative control within group; ^#^ *p* < 0.05 vs. corresponding treatment between groups; ^§^ *p* < 0.05. S, sildenafil.

Given the effects observed at the gene expression level, we set out to evaluate the effect of sildenafil on the intracellular signaling pathways known to be activated by ROS, specifically the phosphorylation/activation status of STAT3, NF-κB, ERK1/2, and AKT in both healthy and SSc cells. In healthy fibroblasts, the pro-oxidant condition induced a 40% increase in p-ERK/ERK (1.4 ± 0.1, *p* < 0.05) and a 30% increase in p-STAT3/STAT3 (1.3 ± 0.0, *p* < 0.05), whereas no differences were observed for p-AKT/AKT and p-NF-κB/NF-κB (*p* > 0.05) (Figure 2A–F). Treatment with sildenafil prevented this upregulation, with levels of p-STAT3/STAT3 and p-ERK/ERK similar to basal values (Figure 2A,C,F). Upon the same pro-oxidant conditions, SSc fibroblasts showed a much greater response in all markers analyzed, with a 320% increase in p-STAT3/STAT3 (3.2 ± 0.2, *p* < 0.05) and 340% increase in p-AKT/AKT (3.4 ± 0.3, *p* < 0.05). Most interestingly, p-NF-κB/NF-κB, which was not affected in healthy cells, showed a 500% increase (51.0 ± 14.8, *p* < 0.05) while p-ERK/ERK was also significantly upregulated (60% increase; 1.6 ± 0.1, *p* < 0.05) (Figure 2B–F). Despite the markedly increased response, treatment with sildenafil reduced the level of almost all protein markers. Specifically, p-STAT3/STAT3 and p-ERK/ERK were completely suppressed (Figure 2A–C,F), whereas pAKT/AKT and p-NF-κB/NF-κB were significantly reduced but remained significantly high compared to either unstimulated conditions or the healthy control group (Figure 2A,B,D,E).

## 3. Discussion

Sildenafil belongs to the class of drugs inhibiting phosphodiesterase type 5 (PDE5i) commonly used to treat erectile dysfunction, Raynaud’s phenomenon, and pulmonary arterial hypertension [15]. PDE5 is a group of ubiquitously present enzymes that hydrolyze cyclic guanosine monophosphate (cGMP) to its inactive form GMP. This cyclic nucleotide plays a prominent role in the regulation of important cellular functions, and PDE5i can therefore elicit a variety of effects [16,17]. The capacity of PDE5i to inhibit cytokine release has been already observed [12,13]. In particular, sildenafil has been shown to have an immunomodulating ability in human immune cells and cardiomyocytes subjected to inflammatory stimuli [12,13]. However, to date, this potential mechanism of action has never been explored in SSc. In this study, we showed for the first time that the PDE5i sildenafil exerts an inhibitory effect on IL-6 and IL-8 gene expression and is released into the culture medium of SSc fibroblasts exposed to ROS. Numerous reports have shown that both IL-6 and IL-8 levels are elevated in culture supernatants of dermal fibroblasts and serum from patients with SSc [10,14]. Consistent with these findings, we observed that SSc fibroblasts cultured in a pro-oxidant environment showed a significant increase not only in IL-6 and IL-8 gene expression, but also their secretion in the medium. It remains to be investigated whether this could be the result of persistent exposure to pro-oxidants and/or of the reduced antioxidant capacity of these cells [9]. Interestingly, sildenafil did not show effects on IL-8 secretion in healthy fibroblasts. In a previous study, performed in patients affected by diabetic cardiomyopathy, we showed that sildenafil could counteract IL-8 release in consequence of a “cut-off” value [13]. Particularly, only patients with a circulating cytokine level above this “cut-off” were responsive to sildenafil treatment with a significant decrease of the chemokine. By contrast, patients with IL-8 below the “cut-off” value were not sensitive to this PDE5i. It is likely that the IL-8 level in healthy fibroblasts was not sufficient to reach the cut-off value, determining a different sensitivity to sildenafil.

As suggested by numerous authors, IL-6 and IL-8 may have a direct effect on regulating tissue fibrosis and endothelial damage [14]. In particular, IL-6 is a pleiotropic pro-inflammatory cytokine capable of stimulating SSc fibroblasts to differentiate and proliferate, causing collagen overproduction and fibrosis [6]. IL-8 is a chemoattractant cytokine responsive to oxidative stress that unlike others has distinct target specificity for neutrophils [18]. The persistent neutrophil activation determines neutrophils accumulation in different body districts (e.g., lung), promoting the genesis of interstitial fibrosis, which is one of the most dreaded clinical manifestations of SSc [7,19]. Indeed, a neutrophil-derived gene signature has been shown to be one of the top discriminants in SSc vs. healthy control blood and a major biological marker of clinical improvement [20]. To begin to dissect the potential mechanism by which sildenafil can modulate IL-8 and IL-6 expression, we analyzed the modulation of proteins such as STAT3, ERK, NF-κB, and PKB/AKT, known to be involved in ROS-mediated signaling. Firstly, we observed a greater modulation of these molecules in SSc compared with healthy fibroblasts, supporting the already proposed notion that SSc fibroblasts may have a reduced ability to counteract the redox-balance [3,21]. Importantly, the presence of sildenafil significantly reduced the phosphorylation levels of these proteins. We believe that, despite not offering a complete explanation, these initial observations do inform and warrant future studies aimed to define the molecular mechanisms underlying this novel biological effect of sildenafil. In this sense, it would be worth exploring the extent to which this effect is directly mediated by cyclic nucleotide hydrolysis inhibition or by independently elevating levels of cAMP and cGMP or modulating ion channels in tissue fibroblasts [22]. In conclusion, we believe that our study, although in vitro and on a limited set of samples, has a strong potential impact. Sildenafil is one of the commonly used drugs in the management of Raynaud’s phenomenon, and given the epidemiological observations strongly indicating that patients with Raynaud’s phenomenon and ANA are at high risk of developing SSc, the dissection of the mechanisms underlying the PDE5i-induced modulation of proinflammatory and profibrotic cytokines following ROS may pave the way to extending the scope of treatment with sildenafil in patients at risk of developing SSc from simple management of Raynaud’s phenomenon to a pre-disease-modifying agent.

## 4. Materials and Methods

### 4.1. Chemicals

DMEM/Ham’s F-12 medium (1:1) with phenol red, phosphate-buffered saline (PBS) Ca^2+^/Mg^2+^-free, Trypsin, bovine serum albumin (BSA), antibiotics, and all reagents for the western blot were obtained from Sigma Aldrich (St. Louis, MO, USA). Fetal calf serum was obtained from Gibco^®^ (US). 2-mercaptoethanol was obtained from Life Technologies, Inc. Laboratories (Grand Island, NY, USA). Hydrogen peroxide (H_2_O_2_), phosphodiesterase type 5 inhibitor (PDE5i), sildenafil citrate salts (S) (98%), and secondary antibodies were purchased from Sigma Aldrich/Merck (Darmstadt, Germany). All reagents for SDS-PAGE were from Santa Cruz (California, USA) and Cell Signaling (Leiden, The Netherlands). For RNA extraction, the TRIzol RNA isolation reagent was purchased from Ambion™; for reverse transcription, 10 mM dNTP Mix, random primers, RNaseOUTNAse™, ribonuclease inhibitor, DNase I^®^ and SuperScript^®^ III Reverse were purchased from Invitrogen. SYBR^®^ Green PCR Master Mix for qPCR was purchased from Life Technologies™ (Applied Biosystems^®^, Waltham, MA, USA). Plastic ware for cell cultures and disposable filtration units for growth media preparation were purchased from Corning (Milan, Italy).

### 4.2. Cell Cultures and Treatments

Human dermal fibroblasts (Hfb) were isolated from excisional skin biopsies from three patients with early diffuse cutaneous SSc (dcSSc) (mean age 61.9 ± 9.2) and three healthy controls (mean age 55.6 ± 8.0) at the SSc clinic within the Leeds Institute of Rheumatic and Musculoskeletal Medicine (UK) and processed as previously described [23]. Informed consent was obtained and approved by the National Research Ethics Service (NRES) Committee (REC 10/H1306/88). Unless otherwise indicated, all cells were treated for 24 h with H_2_O_2_ (100 µM) in the presence or absence of a pre-treatment of sildenafil (1 μM) by adding it to the culture medium 30 min before treatment with hydrogen peroxide. The H_2_O_2_ concentration was selected after dose-response experiments to evaluate cell survival (data not shown). The sildenafil concentration was selected on the basis of the near-therapeutic doses used to treat erectile dysfunction, according to its pharmacokinetics (Cmax and area under the time–concentration curves, AUC).

### 4.3. Cytokine Secretion Assay

Healthy and SSc fibroblasts were plated at 2 × 10^4^ cells/mL in 96-well tissue culture plates. Cell culture supernatants were assayed for IL-6 and IL-8 by magnetic bead-based multiplex assay according to the manufacturer’s protocol. Data acquisition was performed by a Bio-Plex 200 System™ (Bio-Rad Laboratories, Inc., Hercules, CA, USA), which uses Luminex fluorescent bead-based technology. Data analysis was performed by Bio-Plex Manager™ 6.0 software (Bio-Rad Laboratories, Hercules, CA, USA). Cell supernatants were run in triplicate.

### 4.4. RNA Extraction, Reverse Transcription, and Real-Time Quantitative PCR

Total RNA was obtained from ≈3.5 × 10^4^ cells using TRIZOL according to the manufacturer’s instructions and as previously described [24]. Treatment with DNAse enzyme was performed to remove genomic DNA contamination. cDNA was obtained by reverse transcription of 500 ng of total RNA. RT-qPCRs were performed as previously described [25,26,27]. Fluorescence intensities were analyzed using the manufacturer’s software (7500 Software v2.05), and relative amounts were evaluated using the 2−∆Ct method and normalized for ß-actin. Data are expressed as fold increases. Sequences of primers for RT-PCR analysis:

IL-6 Fw: 5′-TTCGGTACATCCTCGACGGC-3′ and Rev: 5′-TCTGCCAGTGCCTCTTTGCT-3′; IL-8 Fw: 5′-TCCTGATTTCTGCAGCTCTGTG-3′ and Rev: 5′-CTGAACCCCAAGGCCAAC-3′; β-actin Fw: 5′-CTGAACCCCAAGGCCAAC-3′and Rev: 5′-AGCCTGGATAGCAACGTACA-3′.

### 4.5. Protein Expression Analysis

Healthy and SSc fibroblasts pre-treated with or without sildenafil (1 µM) and then stimulated for 1 h with 100 µM H_2_O_2_ were lysed in RIPA buffer (150 mM NaCl, 50 mM tris-HCl pH 8, 1 mM EDTA, 1% NP40, 0.25% sodium deoxycholate, 0.1% SDS, water to volume) supplemented with protease and phosphatase inhibitor cocktails (Sigma–Aldrich, Darmstadt, Germany). As previously described [28,29,30], for the immunoblot analysis, an equal amount of proteins (20–30 µg) was resolved in SDS-polyacrylamide (BIO-RAD) gels (10–12%) and transferred onto nitrocellulose membranes (Amersham, Little Chalfont, UK). Thereafter, membranes were incubated with primary antibodies appropriately diluted in Tween Tris-buffered saline (TTBS). Proteins were revealed by the enhanced chemiluminescence system (ECL plus; Millipore). Image acquisition was performed with Image Quant Las 4000 software (GE Healthcare, Chicago, IL, USA) and densitometric analysis with Quantity One^®^ software (Bio-Rad laboratories Inc.). Antibodies utilized were: p-STAT3 (Tyr705), STAT3, p-NF-κB (Ser536) from Cell Signaling; p-AKT (Ser473), AKT, NF-κB, p-ERK1/2 (Thr 44/42), ERK, β actin from Santa Cruz.

### 4.6. Statistical Analysis

All data were generated through experimental triplicates and are represented as the mean ± standard error of the mean (SEM) or as fold increases vs. untreated cells. Protein and mRNA contents were analyzed by one- or two-way ANOVA with Bonferroni’s correction for pair-wise comparisons. Where necessary, a *t*-test analysis was performed. GraphPad Prism 8.0 (La Jolla, CA, USA) was used for all statistical analyses, with significant differences determined by *p* value < 0.05.

## Abbreviations

SSc	systemic sclerosis
RP	Raynaud’s phenomenon
PDE5i	phosphodiesterase type 5 inhibitor
PDEi	phosphodiesterase inhibitors
ROS	reactive oxygen species
IL	interleukin
ANA	Antinuclear Antibodies
Hfb	human dermal fibroblasts
H_2_O_2_	Hydrogen peroxide
